# Isolation and characterization of circulating tumor cells in prostate cancer

**DOI:** 10.3389/fonc.2012.00131

**Published:** 2012-10-11

**Authors:** Elan Diamond, Guang Yu Lee, Naveed H. Akhtar, Brian J. Kirby, Paraskevi Giannakakou, Scott T. Tagawa, David M. Nanus

**Affiliations:** ^1^Division of Hematology and Medical Oncology, Weill Cornell Medical CollegeNew York, NY, USA; ^2^Sibley School of Mechanical and Aerospace Engineering, Cornell UniversityIthaca, NY, USA

**Keywords:** prostate cancer, circulating tumor cells (CTCs), prostate-specific membrane antigen (PSMA), microfluidic device, androgen receptor (AR)

## Abstract

Circulating tumor cells (CTCs) are tumor cells found in the peripheral blood that putatively originate from established sites of malignancy and likely have metastatic potential. Analysis of CTCs has demonstrated promise as a prognostic marker as well as a source of identifying potential targets for novel therapeutics. Isolation and characterization of these cells for study, however, remain challenging owing to their rarity in comparison with other cellular components of the peripheral blood. Several techniques that exploit the unique biochemical properties of CTCs have been developed to facilitate their isolation. Positive selection of CTCs has been achieved using microfluidic surfaces coated with antibodies against epithelial cell markers or tumor-specific antigens such as EpCAM or prostate-specific membrane antigen (PSMA). Following isolation, characterization of CTCs may help guide clinical decision making. For instance, molecular and genetic characterization may shed light on the development of chemotherapy resistance and mechanisms of metastasis without the need for a tissue biopsy. This paper will review novel isolation techniques to capture CTCs from patients with advanced prostate cancer, as well as efforts to characterize the CTCs. We will also review how these analyzes can assist in clinical decision making. **Conclusion:** The study of CTCs provides insight into the molecular biology of tumors of prostate origin that will eventually guide the development of tailored therapeutics. These advances are predicated on high yield and accurate isolation techniques that exploit the unique biochemical features of these cells.

## Introduction

Tumor metastases are a major cause of cancer morbidity and mortality. The precise mechanisms underlying the development of metastases, however, remain poorly understood. Simply stated, this process requires the migration of malignant cells from a primary tumor to distant sites where these cells establish secondary tumors. Circulating tumor cells (CTCs), which were first detected in the blood of an autopsy patient who died from cancer in 1869, are thought to represent tumor cells in transit, some of which will result in metastases (Ashworth, 1869). These cells are capable of intravasation from a primary tumor, undergoing phenotypic alterations that enable intravascular survival, extravasation from the blood vessel, implantation in a target tissue, and proliferation to form a tumor metastasis. Attempts to study CTCs are limited by their rarity, with concentrations as low as one CTC per billion circulating hematopoietic cells. CTCs must therefore be enriched, isolated, and properly identified, in order to be clinically useful. Techniques that exploit the unique physical and biochemical features of CTCs are currently being developed and utilized in order to isolate and identify CTCs from whole blood samples obtained from cancer patients. Currently, there are numerous techniques available to detect and isolate CTCs (Table 1). With the exception of the CellSearch Circulating Tumor Cell Test, these techniques have not yet been approved by the Food and Drug Administration (FDA) for clinical use. CTC enumeration using the CellSearch device has already been shown to correlate with patient outcomes in a variety of malignancies, including prostate cancer (Danila et al., 2007). Capture technologies may also provide rare opportunities to perform molecular and genetic analyses of tumor-derived cells at sequential time points without invasive tissue biopsies. Thus, CTCs conceptually provide insight into the biology of a patient's tumor that may facilitate the development of new therapeutic options and enable clinicians to tailor therapy to an individual patient in a longitudinal fashion (van de Stolpe et al., 2011). It follows that CTC isolation can replace biopsies and noninvasively yield valuable information about the evolving status of a patient's disease.

**Table 1:** **Summary of techniques used to isolate prostatic CTCs**.

**Method**	**Mechanism**	**Volume of blood used (ml)**	**Capture rate**	**References**
Density gradient centrifugation	Differential migration of CTCs during centrifugation	Variable	70%	Rosenberg et al., 2002; Gertler et al., 2003; Kuhn and Bethel, 2012
Size-dependent selection	Separation based on cell diameter	6–7.5	90%	Vona et al., 2000; Lin et al., 2010; Farace et al., 2011
Immunomagnetic bead-based capture (CellSearch)	Positive selection using EpCAM coated magnetic beads	7.5	85%	Tibbe et al., 2002; Allard et al., 2004; Balic et al., 2005
Antibody-based negative selection	Depletion of normal blood cells using CD-45 coated magnetic beads	2.5 ml	52–88.4%	Wang et al., 2000; Zigeuner et al., 2000, 2003; Jatana et al., 2010; Liu et al., 2011; Schmidt et al., 2004
Flow cytometry	Cell sorting using fluorescently labeled epithelial antigens	NA	NA	Racila et al., 1998; He et al., 2008; Wu et al., 2011
Microfluidic device	Positive selection of CTCs using antibodies attached to microfluidic device	1–5.1	60–91.8%	Nagrath et al., 2007; Gleghorn et al., 2010; Stott et al., 2010a,b; Mayer et al., 2011; Kirby et al., 2012; Santana et al., 2012

Analyzing peripheral blood is an attractive alternative to currently available methods of obtaining tissue in prostate cancer owing to the unique challenges presented by this disease. A man with prostate cancer may not develop metastases until many years (5–15 years) after treatment of his original tumor in the prostate. Thus, performing a molecular analysis of archived prostate cancer tissue may be complicated by the inability to obtain old pathology specimens and by the possible irrelevance of that tissue sample to the current status of the patient's disease. Ideally, a tumor biopsy for molecular study would be obtained at the time of relapse, but as many men have only bone metastases, it is difficult to obtain adequate and representative tumor cells for study. In a disease for which a blood test measuring prostate specific antigen (PSA) is sufficiently specific to support the diagnosis of prostate cancer, it is difficult clinically to justify a biopsy. Consequently, analysis of peripheral blood overcomes these obstacles by easily providing clinically relevant tumor cells for study.

Ideally, a robust CTC capture technique would be highly sensitive, specific, reproducible, and automated (Doyen et al., 2012). It should have the ability to reliably capture a high percentage of CTCs present in a sample while minimizing the number of false positive events and contamination from non-malignant cells. The design of the test should be simple enough that it can be mass-produced and be performed in clinical laboratories with minimal inter-operator variability. It should also have the ability to both quantify and characterize CTCs in order to limit operator bias. Most importantly, in order to be clinically useful, a CTC capture technology should have proven clinical relevance confirmed in multiple prospective clinical trials. In this chapter, we will review the currently available CTC enrichment technologies with an emphasis on prostate cancer as well highlight current and future applications of these technologies.

## CTC detection methods

Accurate characterization of CTCs is essential to the development of these cells as a clinical biomarker and substrate for laboratory experimentation. There is currently, however, no “gold standard” approach for the specific identification of CTCs. This is essential, in part, because most available CTC enrichment technologies yield samples composed of hematopoietic cells, CTCs, and, in some cases, benign epithelial cells. Genomic analysis and surface antigen detection are the two most commonly used methods for CTC detection. Reverse transcription polymerase chain reaction (RT-PCR) and Fluorescence *in situ* hybridization (FISH) have been used to identify tumor-specific genetic and chromosomal features in order to differentiate CTCs from contaminating cells. Immunofluorescent microscopy is utilized to detect epithelial specific antigens such as epithelial cell adhesion molecule (EpCAM) or cytokeratin (CK), or prostatic antigens such as PSA and prostate-specific membrane antigen (PSMA).

### Polymerase chain reaction

Reverse transcription-PCR is highly sensitive for identifying the presence of CTCs and is able to detect a single malignant cell among ten million peripheral blood mononuclear cells (PBMCs) (Gomella et al., 1997). In addition to its sensitivity, RT-PCR has the potential to detect mRNA from CTC fragments that may otherwise not be detected through direct visualization by immunohistochemistry (Sun et al., 2011). This technology has been used in various ways to detect CTCs. In early experiments, CTC capture was performed on whole blood samples to detect tumor-specific genes. Extracellular RNA is highly unstable and its presence in peripheral blood suggests the existence of circulating cells expressing tumor-specific transcripts (Seiden et al., 1994). For instance, detection of circulating prostate-specific RNA transcripts for PSA or PSMA is thought to indicate the presence of prostatic CTCs. The first study to detect CTCs from venous blood samples using RT-PCR was performed in 1992 by Moreno et al. (1992). They identified PSA mRNA in blood samples from 4 of 12 patients with metastatic prostate cancer and in none of the 17 controls, including subjects with benign prostatic hypertrophy (Moreno et al., 1992). Subsequent studies of PCR in prostate cancer have utilized PSMA, kallikrein-2 (hK2), and PTI-1, in addition to PSA, as prostate-specific markers (Olsson et al., 1997; Kurek et al., 2004).

There are several potential limitations to RT-PCR. It suffers from poor specificity, as it may detect target RNA shed by normal prostatic cells. Furthermore, “illegitimate transcripts,” tissue-specific genes that are expressed such as spliced transcripts in non-specific tissues, may also lead to false positive results (Chelly et al., 1989; Zippelius and Pantel, 2000). For example, in a quality-control study, PSA and PSMA were detected in non-prostatic negative control cell lines and healthy donor blood, which upon further analysis were found to be perfectly homologous with the exception of specific sequence deletions or point mutations not found in RNA transcripts native to prostatic tissue (Gala et al., 1998).

This issue has been addressed in part by the introduction of quantitative PCR (Q-PCR), which increases the specificity of mRNA detection by use of transcript-specific probes and enables the determination of mRNA copy number such that above a specific threshold a transcript is thought to be of malignant origin (Pantel et al., 2008). In one study, PSA mRNA copy number used as a surrogate for CTC count was predictive of recurrence after radical prostatectomy (Yates et al., 2012). Several studies have shown significant differences in PSA and PSMA mRNA copy number among patients with benign prostatic hypertrophy, localized prostate cancer, and metastatic disease (Zhang et al., 2008; Kalfazade et al., 2009). A study using Q-PCR for Kallikrein-2 (klk-2), PSA, and prostate specific stem cell antigen (PSCA) mRNA, copy number was concordant with CellSearch Circulating Tumor Cell Test CTC counts, and were predictive of metastatic disease vs. localized prostate cancer. It should be noted that there was 95% concordance for patients with more than 15 CTCs but diminished significantly for CTC counts less than 5 cells per 7.5 ml of blood (Helo et al., 2009).

Nevertheless, the PCR approach has many potential drawbacks. Expression of target RNA markers varies significantly between patients and among different tumor cells derived from the same patient, complicating the interpretation of absolute RNA copy number. Additionally, false negative results may occur because of low levels of target RNA expression in patients who have CTCs and metastatic disease. Furthermore, this technique is not able to distinguish between viable and non-viable CTCs. Finally, PCR does not allow for the direct visualization of CTCs and further molecular analysis using other laboratory techniques (Sun et al., 2011).

### Surface marker detection

**Figure 1:**
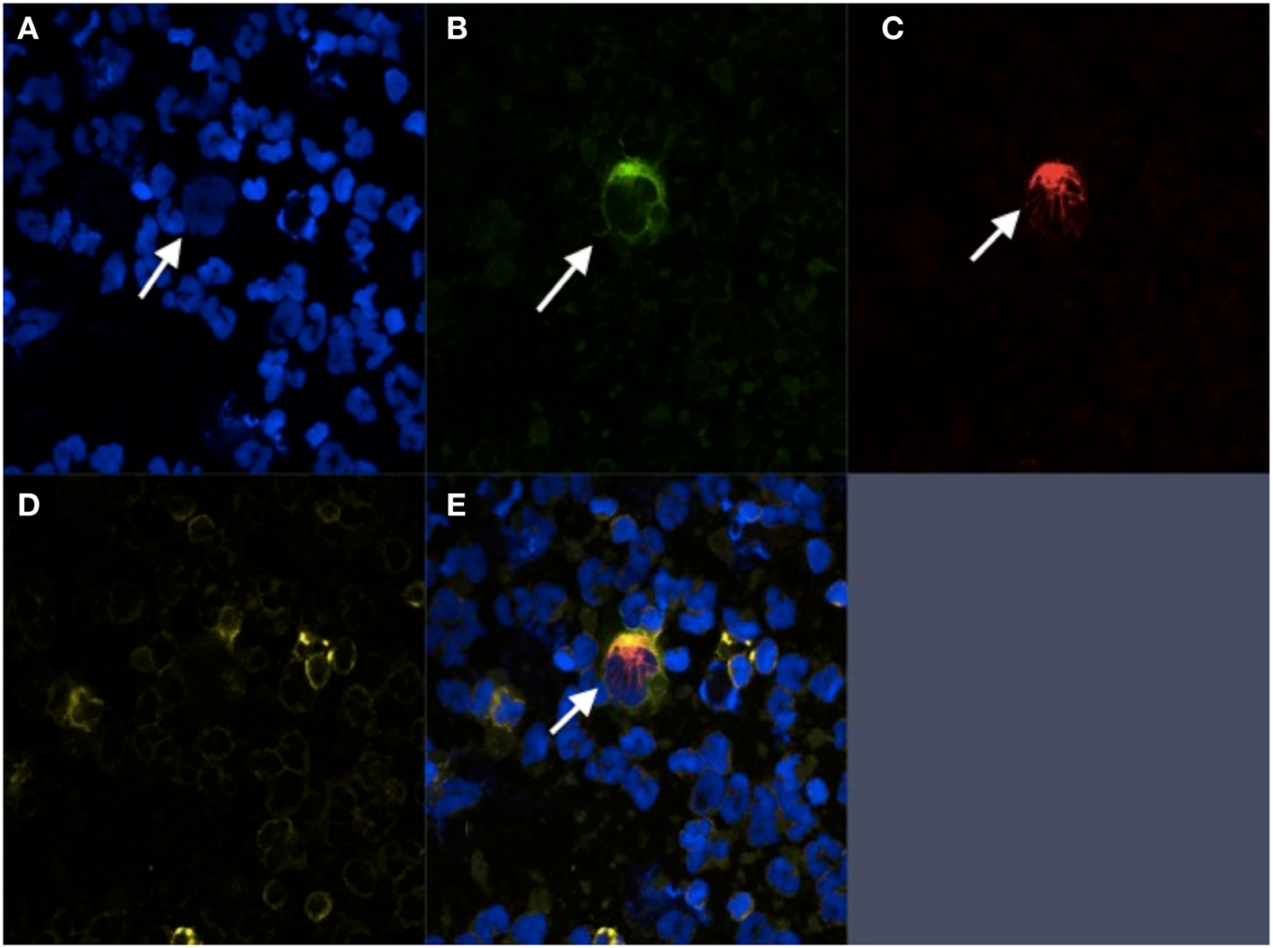
**Immunofluorescent staining of prostate cancer CTC.** CTC isolated from patient with CRPC using negative selection. **(A)** DAPI; **(B)** PSMA; **(C)** Cytokeratin; **(D)** CD-45; **(E)** Composite image.

Immunofluorescent staining is one of the most widely used methods of identifying CTCs enriched from heterogeneous cell populations (Figure 1). This allows for direct visualization of cells by fluorescent microscopy and for discrimination of CTCs from surrounding leukocytes by differential antigen expression. Nuclei are identified using DAPI, a fluorescent molecule that binds to the adenine- and thymine-rich regions of DNA (Zink et al., 2003). Anti-EpCAM and anti-CK antibodies are then used to confirm the cells are of epithelial origin. Leukocytes are differentiated from epithelial cells by the presence of CD45, a tyrosine phosphatase that is expressed on hematopoietic cells. Using common platforms such as CellSearch, a cell is said to be a CTC if it is DAPI positive, stains positively for CK or EpCAM, and stains negatively for CD45 (Allard et al., 2004). Interestingly, cell populations co-expressing epithelial markers and CD45 have been detected using CellSearch and other CTC isolation technologies. The significance of these cells is poorly understood and these cells are currently excluded from enumeration (Yu et al., 2011).

Antibodies directed against PSA and PSMA provide additional specificity to immunofluorescent identification of prostatic CTCs (Wang et al., 2000; Stott et al., 2010b). As mentioned previously, PSMA is a non-secreted protein expressed in prostatic tissues and to a much lesser extent, non-prostatic cell types such as renal tubular cells and intestinal epithelial cells (Troyer et al., 1995; Bostwick et al., 1998; Sweat et al., 1998; Sokoloff et al., 2000). Its expression is significantly upregulated on prostate cancer cells and is also seen in the neovasculature of the majority of solid-organ malignancies. PSA is a kallikrein found in high concentrations in prostatic cells and seminal tissues, and to a lesser degree in non-prostatic tissue types such as mammary, lung, and uterine tissue (Wei et al., 1997; Fortier et al., 1999; Mannello and Gazzanelli, 2001). Other fluorescently labeled antibodies may also be employed to detect subcellular localization of proteins of interest. For example, antibodies that recognize androgen receptor (AR) and tubulin have been used in prostate cancer CTCs to determine changes in the distribution of these proteins in the presence of androgens before and after taxane treatment to determine susceptibility to these agents (Darshan et al., 2011).

Several different prostatic CTC morphologies have been identified using immunofluorescent microscopy. Large cells with irregularly shaped nuclei are the predominant CTC cell type. Other cell morphologies include very large fragile cells with loose chromatin, CK- and PSMA-positive enucleated cells, cellular debris, stem cell-like cells, and micro-clusters composed of 3–100 CTCs. The significance of these different morphologies is uncertain but may represent two distinct populations of cells; one which has no reproductive ability, and the other with growth potential and consequently metastatic potential (Wang et al., 2000). Of note, although the biological significance of CTC fragments is unknown, there is also evidence that enucleated CTCs and CTC fragments correlate with patient outcomes in prostate cancer (Coumans et al., 2010).

In addition to immunofluorescence, flow cytometry has been frequently used to detect prostatic CTCs on the basis of surface antigen expression (Racila et al., 1998; He et al., 2008). In one study, a fluorescently labeled phosphoramidate peptidomimetic PSMA inhibitor was used to detect PSMA positive cells with flow cytometry (Wu et al., 2011). The authors found that there was reasonable concordance between the number of cells spiked in a sample and the number determined by flow cytometry (Wu et al., 2011). Prostate cancer CTCs isolated by flow cytometry cell sorting can also be analyzed by multiplex RT-PCR for expression prostate-specific mRNAs such as PSA, AR, and the prostate cancer specific gene fusion TMPRSS2 (Danila et al., 2011).

### Fluorescence *in situ* hybridization

Visual detection of tumor-specific genomic material is an alternate means of detecting and characterizing CTCs after enrichment with the added benefit of providing potentially clinically useful information. FISH is technique that uses fluorescent nucleic acid based probes that hybridize with genes of interest that are visualized using fluorescent microscopy. Several studies have successfully employed FISH to detect prostatic CTCs from enriched blood samples. In one study, enumerator probes designed to detect chromosomal aneusomy typical of prostatic malignancies identified prostatic CTCs in samples enriched using anti-EpCAM coated immunomagnetic beads. Interestingly, the authors found concordance between the chromosomal abnormalities detected in CTCs with those found in the primary tumor in a significant proportion of cases, supporting the theory that these cells are indeed tumor derived (Fehm et al., 2002). FISH probes have been used to detect AR amplification, gain of the MYC oncogene, and loss of the 8p gene locus in CTCs enriched using the CellSearch Circulating Tumor Cell Test. FISH using these probes also demonstrated that prostatic CTCs have similar cytogenetic profiles to advanced prostatic tumors, a finding that is consistent with data correlating higher CTC counts with poor clinical outcomes (Leversha et al., 2009).

## CTC enrichment methods

### Density-dependent enrichment

Density-gradient centrifugation separates CTCs from whole blood based on the differential migration of cells through a fluid in a density-dependent manner during centrifugation. Whole blood centrifuged using a density gradient solution such as ficoll-paque™ separates blood into a layer of plasma, PBMCs, and an anucleate cell layer composed of erythrocytes and platelets. As mononuclear cells, CTCs migrate to the PBMC layer, which may be isolated for further processing. The advantages of this technique are that it is relatively inexpensive, easy to perform, and yields intact CTCs that can be subjected to further experimentation. Perhaps most importantly, it enables the capture of CTCs without relying on the expression of epithelial-specific surface markers commonly used in positive selection techniques (Sun et al., 2011). Under optimal conditions, density gradient centrifugation is able to capture ~70% of CTCs present in a sample. The remaining cells are likely lost in the plasma or anucleate cell layer. The negative aspect of this approach is that samples obtained through this method are impure and are overwhelmingly composed of hematopoietic mononuclear cells. This makes the detection of CTCs using immunohistochemistry both difficult and time consuming. A newer density gradient solution, Oncoquick™, which employs a porous membrane, has been shown to prevent cross-contamination between layers and to improve sample purity (Rosenberg et al., 2002; Gertler et al., 2003). Nevertheless, because samples processed in this manner have significant leukocyte contamination, density gradient centrifugation is most often used as a precursor to other CTC enrichment procedures such as PCR-based and negative selection techniques.

A variation of density-gradient centrifugation is to use high-density imaging following isolation and immunostaining to identify CTCs using multiple fluorescent channels to produce high quality and high resolution digital images that retain fine cytologic details of nuclear contour and cytoplasmic distribution (Marrinucci et al., 2012). This enrichment-free strategy results in high sensitivity and high specificity, but still lacks the ability for further molecular analysis of identified CTCs.

### Size-dependent selection

In general, CTCs that emanate from solid tumors have a larger diameter and volume than other hematological cells found in the circulation. Consequently, many investigators have tried to exploit this characteristic in designing approaches to capture CTCs. The most common approach is to use a filtration-based device in which whole blood is passed through a filter with an 8 μm pore diameter, enabling the passage of most hematopoietic cells while retaining larger cells such as CTCs. Isolated cells are then stained for epithelial surface markers in order to identify the CTC population. This method, entitled ISET, has a high capture efficiency for cells >8 μm in diameter, which ranges between 86% and 100% of CTCs. It is sensitive enough to isolate a single micro-pipetted tumor cell added to one milliliter of blood and yields CTCs that are amenable to further experimentation such as PCR and flow cytometry (Vona et al., 2000; Zabaglo et al., 2003; Lin et al., 2010). In one study, a portable filter-based device achieved 90% capture efficiency from blood spiked with a prostate cancer cell line and found that it enriched more prostatic CTCs from more patient samples than did the FDA-approved CellSearch device (Lin et al., 2010). A prospective trial of 60 patients, 20 of whom had PC, further established ISET's sensitivity for detecting prostatic CTCs when compared with CellSearch (Farace et al., 2011). This approach has several practical advantages; it is relatively inexpensive and easy to perform (Lin et al., 2010). Furthermore, it does not rely on surface marker expression, which may vary widely, leading to inefficient cell capture. Filtration-based devices, however, may lack sensitivity when used to isolate CTCs from patient blood samples. Cell lines used to assess sensitivity and specificity of these devices tend to be composed of homogeneous, large tumor cells that may be consistently captured using this system. Patient-derived CTCs are heterogeneous and may not be large enough to be enriched. Thus, size-dependent filtration may underestimate the true number of CTCs in a given patient's blood (Wang et al., 2000; Stott et al., 2010b). Pore size may also limit the specificity of ISET-based devices, as certain classes of hematopoietic cells, such as neutrophils, plasma cells, and macrophages, are larger than 8 μm in diameter. Additionally, although most lymphocytes are 7–8 μm in diameter, larger lymphocytes may be captured, further limiting the specificity of this technology.

### Negative selection by use of immunomagnetic beads

CTCs isolated from the mononuclear cell layer generated by density gradient centrifugation can be further purified using ferromagnetic anti-CD45 coated beads (Zigeuner et al., 2003). CD45 is a protein tyrosine phosphatase that is present on all hematopoietic cells with the exception of plasma cells and erythrocytes and is typically not expressed in epithelial cells (Stelzer et al., 1993). CTCs are negatively selected by depleting CD45-positive cells from a blood sample. Cells that bind to the beads are separated from the sample using a magnetic field. This technique has a reported capture efficiency ranging from 52% to 88%, but still has many of the limitations of density gradient centrifugation. The probability of isolating one cell spiked into one million leukocytes is 93.3% (Wang et al., 2000; Zigeuner et al., 2000). This technique has been used to detect cells in a variety of malignancies including prostate cancer (Wang et al., 2000; Schmidt et al., 2004; Yang et al., 2009). In one study, negative selection was used to isolate CTCs in patients with metastatic prostate cancer with a PSA decline while undergoing cytotoxic chemotherapy, demonstrating that CTCs may be present despite evidence of biochemical response to chemotherapy (Schmidt et al., 2004). A major advantage of this technique is that it does not rely on the expression of tumor-specific markers, enabling capture of cells that would otherwise be missed by positive selection methods (Liu et al., 2011). Negative selection also yields intact CTCs that are amenable to further experimentation. Samples isolated using this technique, however, still suffer from a lack of purity because not all CD45-positive cells are removed during sample processing. Because this process requires density gradient centrifugation, it also lacks sensitivity owing to the loss of cells in plasma or RBC layers. Additionally, negative selection by use of CD45-coated beads also adds several washing steps that may further contribute to low capture efficiencies.

### Positive selection by use of immunomagnetic beads

Ferromagnetic beads are also used to positively select for prostate cancer CTCs by exploiting their expression of epithelial cell-surface antigens. Cells isolated during density gradient centrifugation are incubated with anti-EpCAM and anti-CK coated magnetic beads, which bind to CTCs and remove them from the sample when a magnet is applied (Brandt et al., 1996; Jost et al., 2010). EpCAM is a type I membrane protein that functions as a cell adhesion molecule in epithelial and adenomatous cell types and is highly overexpressed in various carcinomas including prostate cancer (Litvinov et al., 1996; Mukherjee et al., 2009). CK is an intermediate filament component of the cytoplasm of epithelial cells and, to a lesser degree, in non-epithelial cell types including smooth muscle and endothelial cells (Franke et al., 1979; Mattey et al., 1993). In 2000, Wang et al. described isolation of CTCs from peripheral blood with centrifugation density gradients and magnetic cell sorting (Wang et al., 2000). This technology has evolved and today capture devices utilizing this approach are one of the most extensively studied methods of enriching CTCs. The CellSearch Circulating Tumor Cell Test device, which is the only FDA-approved test for CTC enrichment, positively selects CTCs from 7.5 ml of whole blood using EpCAM coated magnetic beads, separates them from other blood components using a magnetic field, and immunofluorescently labels them with 4′,6-diamidino-2-phenylindole (DAPI), anti-CD45 and anti-CK antibodies. A computer screen displays presents an operator with images of cells for review and enumeration (Tibbe et al., 2002). This method is 85% sensitive for the detection of cultured breast cancer cells spiked into whole blood (Riethdorf et al., 2007). This device has been shown to have a low false-positive rate in a series of 2,183 patients with metastatic cancers; CTCs were detected in 36% of patient samples and 0.3% of healthy controls (Allard et al., 2004). In the subset of patients with metastatic prostate cancer, more than two CTCs were detected in 37% of patients (Allard et al., 2004). It has also been shown to be more sensitive and specific than density-dependent centrifugation with Oncoquick™ (Balic et al., 2005). Cells captured from patients with metastatic prostate cancer by use of this device have also been shown to possess other molecular features of prostate cancer cells such as AR gene amplification (Shaffer et al., 2007; Attard et al., 2009).

Despite multiple studies validating the CellSearch system's prognostic value as related to CTC enumeration, several important caveats limit its usefulness. It is both expensive and time consuming to perform. The CellSearch device fixes cells prior to staining them, significantly limiting the ability to perform subsequent functional assays and nucleic acid analysis (Stott et al., 2010b). Most importantly, the sensitivity of this device is limited by its use of EpCAM-based detection (Lara et al., 2004). CTCs express variable levels of EpCAM *in vivo*, due, in part, to downregulation of epithelial surface markers. This process, known as epithelial-to-mesenchymal transition (EMT), is a process in which epithelial CTCs assume a mesenchymal phenotype in preparation for extravasation and implantation in metastatic sites (He et al., 2010; Armstrong et al., 2011). These cells are more likely to metastasize and have been linked to more aggressive prostate cancers in a number of clinical studies (Tomita et al., 2000; Gravdal et al., 2007). Evidence for EMT has been found in CTCs that express both epithelial markers such as CK and EpCAM and mesenchymal markers such as vimentin, e-cadherin, and CD133 (Armstrong et al., 2011). The co-expression of these markers is thought to represent an intermediate state between the two cell types (Armstrong et al., 2011). CTCs that undergo EMT are less likely to express high levels of EpCAM and may therefore evade capture by anti-EpCAM antibodies (Santana et al., 2012). The superior capture efficiency of non-EpCAM based capture technologies such as ISET and PSMA microfluidic devices may, in part, be explained by this phenomenon.

### Microfluidic devices

Microfluidic devices have demonstrated high capability to enrich CTCs from whole blood. One example, the “CTC-chip” is composed of an array of antibody-coated microscopic posts arranged as equilateral triangles through which a blood sample is flowed. As described, the arrangement of the posts is designed to minimize the shear forces that cells are exposed to while within the device. CTCs within the sample collide with the posts and are specifically captured by the antibodies used to coat them (Nagrath et al., 2007). In 2007, Nagrath et al. successfully employed a CTC-chip functionalized with anti-EpCAM antibodies to isolate CTCs from whole blood taken from patients with a range of epithelial malignancies. Capture efficiency of approximately 60% was determined by spiking blood from healthy donors with a non-small cell lung cancer cell line. Interestingly, capture efficiency was not diminished by using cell lines with low levels of EpCAM expression. The authors were able to identify CTCs in 115 of 116 (99%) samples taken from patients with breast, colon, pancreatic, or prostate cancer with an average purity of 49–67%. Similar to the CellSearch Circulating Tumor Cell Test device, in a limited analysis, the authors were able to correlate patient outcomes and response to treatment with the number of CTCs captured (Nagrath et al., 2007).

The same investigators developed what they term a “herringbone chip” to use the vortical flow induced by anisotropic surface grooves to generate a device exhibiting chaotic advection (Stroock et al., 2002). The device consists of eight micro-channels etched with periodically occurring herringbone-shaped grooves and functionalized with anti-EpCAM monoclonal antibodies. The herringbone device has a capture efficiency of 91.8% +/−5.2% in cell spiking experiments using PC-3 cells, and CTCs were detected in 93% of samples from patients with metastatic prostate cancer. The design of the herringbone device chip enabled cell capture at 50% more efficiency than the post-based anti-EpCAM device from the same investigators. Captured cells are again amenable to further experimentation such as PCR and on-chip immunofluorescent staining (Stott et al., 2010a).

**Figure 2:**
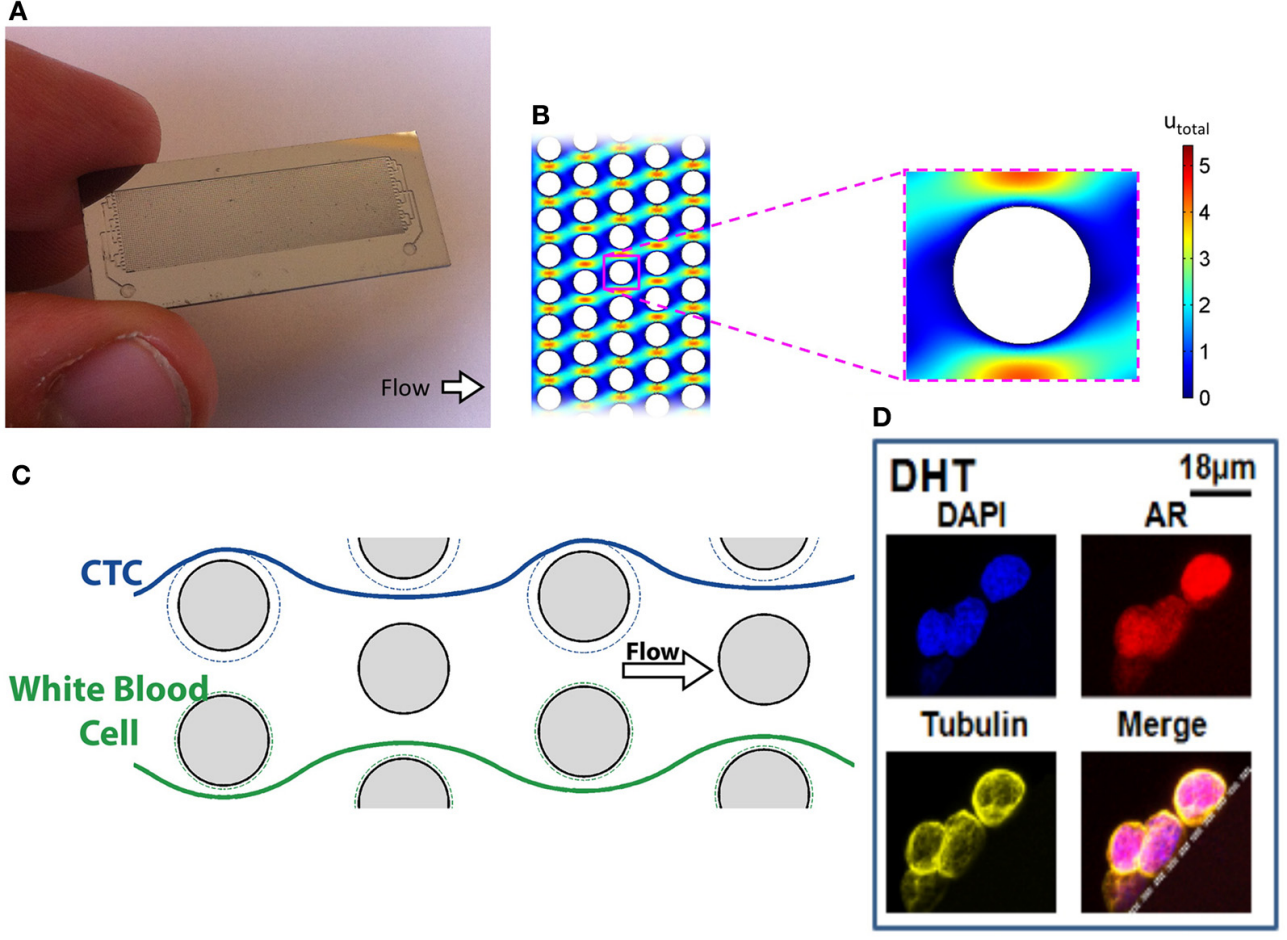
**Geometrically-enhanced differential immunocapture (GEDI) microfluidic device. (A)** GEDI Chip **(B)** GEDI post-array **(C)** Illustration of laminar flow through GEDI device **(D)** Captured CTCs stained for AR and tubulin.

Despite showing effective capture using cell lines with low levels of EpCAM expression, it is unclear whether chips functionalized with anti-EpCAM antibodies can efficiently capture cells that have undergone EMT in patient samples. CTCs may express lower levels of EpCAM than cultured cells used for these experiments and may evade capture. PSMA based CTC capture may be able overcome this limitation in prostate cancer patients and enable the capture of CTCs that have undergone EMT. PSMA, also known as glutamate carboxypeptidase II, is a type II transmembrane metallopeptidase that is universally expressed in prostatic tumors and may be conserved during EMT. Furthermore, levels of expression correlate with disease severity, suggesting utility as a prognostic marker (Bostwick et al., 1998; Sweat et al., 1998). Although it is normally expressed as a cytoplasmic protein in benign prostatic cells, alternative splicing of PSMA mRNA in prostatic carcinomas leads to its expression as a type II integral surface membrane protein, making it a suitable target for anti-PSMA antibody based capture (Israeli et al., 1993). Although this marker is not entirely specific to prostatic cells, expression in this population is 100–1000 times greater than cells in other tissue types such as cells of the small intestine, proximal renal tubules, and salivary glands (Troyer et al., 1995; Sokoloff et al., 2000). The J591 antibody is a deimmunized monoclonal antibody that specifically recognizes an extracellular epitope of PSMA. This antibody has been used successfully to capture CTCs from whole blood using a geometrically-enhanced differential immunocapture (GEDI) microfluidic device (Figure 2) (Gleghorn et al., 2010). The PSMA-GEDI “chip” is designed to maximally increase the frequency of CTC collisions with anti-PSMA immunocoated posts in a size and flow-dependent manner. Size-based selection is thought to increase capture efficiency and improve purity by limiting opportunities for non-target blood cells to interact with the immunocoated surfaces. The capture efficiency of the PSMA-coated GEDI chip is quite high, 97 ± 3% for cells spiked in PBS and 85 ± 5% for cells spiked in whole blood (Gleghorn et al., 2010).

## Current and future applications of CTC enrichment devices

The FDA approved the CellSearch device for monitoring disease status in patients with metastatic prostate cancer (Wang et al., 2011). Studies using this device have demonstrated that prostate cancer patients with at least 5 CTCs in 7.5 ml of blood have an inferior overall survival compared with patients with less than 5 CTCs in 7.5 ml (Danila et al., 2007). The IMMC38 trial, which provided the basis of FDA clearance of the CellSearch device in prostate cancer, reported that a CTC count greater than 4 cells/7.5 ml is associated with unfavorable response to therapy in metastatic castrate-resistant prostate cancer patients (Scher et al., 2009). Several subsequent studies did not detect a threshold effect; suggesting the use of CTC counts as a continuous variable without a specific cutoff value (Danila et al., 2007). Nevertheless, chemotherapy-naïve patients with CTC counts greater than 4 cells/7.5 ml have a 45% decrease in overall survival when compared to those with fewer than 5 CTCs. The impact of CTC counts is even greater in patients who had undergone one or more chemotherapeutic regimens, where patients had a 60% decrease in overall survival (Danila et al., 2007). CTC counts are also useful in patients with hormone-sensitive PC. A CTC cutoff of three or more cells, detected using the CellSearch device was able to predict the magnitude and duration of response to androgen deprivation therapy in these patients (Goodman et al., 2011). Studies also compared CTC counts with traditional markers of disease progression and found that it is a more powerful predictor of survival and therapeutic response than currently used biomarkers such as PSA (de Bono et al., 2008; Scher et al., 2009).

The number of prostate cancer CTCs has also been studied as a secondary endpoint in a number of clinical trials. Two recent phase II trials examined the efficacy of abiraterone acetate, a CYP17 inhibitor that impairs androgen synthesis, in patients with castration resistant prostate cancer used CTCs as efficacy markers. In one study, CTCs were isolated from patient blood by use of the CellSearch Circulating Tumor Cell Test prior to treatment and every 4 weeks during treatment. The authors found significant declines in CTC counts of treated patients, with 63% of patients having a greater than 50% decrease in CTCs. This decline mirrored the PSA decline in a subset of patients with ERG gene mutations (Reid et al., 2010). A second study, which aimed at defining the efficacy of abiraterone combined with prednisone in metastatic castrate-resistant prostate cancer patients who failed first line chemotherapy used conversion from unfavorable to favorable CTC counts as a surrogate of clinical response. The authors reported that 34% of treated patients who had pre-treatment CTC counts greater than 5 cells/7.5 ml had a decrease in CTC counts to less than 5 cells/7.5 ml (Danila et al., 2010). Several recently reported and ongoing phase III studies are validating this biomarker as a potential surrogate marker of response and survival (Scher et al., 2011).

Although still in its early stages, molecular and genetic analyses of CTCs have also been used to correlate CTC characteristics with treatment outcomes. For example, a study using FISH to detect the fusion gene TMPRSS2-ERG demonstrated a significant association between expression of this marker and PSA response to abiraterone. Furthermore, this study also demonstrated a high degree of concordance between the presence of the fusion gene in CTCs and in primary prostatic tumors, further supporting the utility of CTCs as a “liquid biopsy” (Attard et al., 2009). A second study examining TMPRSS2-ERG mRNA in CTCs showed no relationship to patient outcomes (Gopalan et al., 2009; Fine et al., 2010).

Studies have also examined the predictive value of nuclear and/or cytoplasmic localization of AR in CTCs. The AR plays a key role in the development and progression of prostate cancer. In hormone-sensitive prostate cancer, systemic androgens induce AR-mediated cellular proliferation, which is impaired by androgen deprivation therapy by preventing ligand-dependent nuclear AR translocation. AR signaling can continue to stimulate tumor growth in castrate patients via intra-tumoral androgen synthesis or constitutive AR activation-independent of ligand binding (Chen et al., 2004). Recent studies suggest that taxane chemotherapy in prostate cancer can impede AR translocation from the cytoplasm to the nucleus by disrupting microtubules that normally function to transport AR to the nucleus (Darshan et al., 2011). In a pilot study of patients receiving taxane chemotherapy, examination of AR nuclear localization and microtubule integrity in CTCs isolated using the CellSearch Circulating Tumor Cell Test device correlated with response to therapy (Darshan et al., 2011). In an unrelated study, PCR based analysis of prostate cancer CTCs detected several AR mutations some of which have been associated with therapeutic resistance to anti-androgen therapy (Jiang et al., 2010). Recent studies have also shown that AR splice variants may evolve with therapy and be a mechanism of treatment resistance (Sun et al., 2010; Guo and Qiu, 2011; Mostaghel et al., 2011). Studies are in progress to determine if these abnormalities in AR that could affect treatment decisions can be detected by examining CTCs.

## Conclusion

The science of CTC capture and analysis is evolving and will certainly change as newer technologies are incorporated and validated. The only FDA-cleared device, CellSearch system, has been shown to be an important prognostic tool, providing valuable insights into treatment response and overall survival. Experiments with alternative enrichment methods highlight the poor sensitivity of the CellSearch technique, with multiple studies demonstrating significantly higher capture rates from patient with metastatic castrate-resistant prostate cancer. However, their clinical utility remains to be confirmed. Further studies are needed to improve and validate alternative enrichment in identification techniques.

The effect that CTC analysis will have on patient care remains to be determined. As discussed, genetic analysis of CTCs has enabled the detection of abnormalities that influence tumor sensitivity to a variety of prostate cancer therapies. Molecular analysis has helped elucidate the mechanism of taxane anti-tumor effect in prostate cancer, and provides a basis for an assay to assess the likely efficacy of this chemotherapeutic class. Future studies will be aimed at assessing additional markers of treatment sensitivity and resistance, and attempting to ascertain additional drug targets. Additional studies correlating the molecular features of CTCs with those of tissue specimens obtained from primary and metastatic sites are needed. The ultimate goal is to develop technology that will enable periodic monitoring of tumor biology in a way that will enable clinicians to effectively tailor therapy to the individual patient on an ongoing basis in order to maximize patient outcomes.

### Conflict of interest statement

The authors declare that the research was conducted in the absence of any commercial or financial relationships that could be construed as a potential conflict of interest.
